# 

**Published:** 1862-01

**Authors:** 


### DENTAL REGISTER

### OF THE WEST.

EDITED BY

J. TAFT AND GEO. WATT.

JANUARY—1862.

**MONTHLY:**

**Three Dollars per Annum, in advance.**

CINCINNATI:
J. T. TOLAND, PUBLISHER AND PROPRIETOR.

J. TAFT, DENTIST, No. 56 WEST FOURTH STREET.

